# Supplementary material for: Quantitative characterization of 3D bioprinted structural elements under cell generated forces
Source: Nat Commun. 2019 Jul 10;10:3029. doi: 10.1038/s41467-019-10919-1 (PMC6620298; doi:10.1038/s41467-019-10919-1)
Supplement: Supplementary file 1 — Supplementary Information [file 41467_2019_10919_MOESM1_ESM.pdf]

Quantitative Characterization of 3D Bioprinted Structural Elements Under Cell Generated Forces

Morley and Ellison et al.

Supplementary Information

Printing Parameters and Performance

Our previous work and our analysis of the 3D printing literature suggest that the cross-sectional area, *A*, of 3D printed cell-ECM microbeams can be controlled by varying two independent variables: the volumetric flow-rate, *Q*, at which ink is deposited into the microgel medium, and the translation speed, *v*, of the printing needle tip[1,2]. These three parameters generally obey a continuity equation that predicts the approximate cross-sectional area of printed filaments, given by $Q = vA$. To test whether this relationship holds for 3D printing cell-loaded ECM microbeams, we perform prints at many combinations of *Q* and *v*, with *Q* spanning 10 – 60 μL/hr, and *v* spanning 0.1 – 0.5 mm/s. We measure the cross-sectional area of these beams with 3D confocal microscopy scans and image analysis. Approximating the cross-sections as circular, we measure an effective diameter, D_m, which correlates excellently with the predicted diameter, D_p ($R^2 = 0.96$), following the relationship $D_m = 0.83D_p$ (Supplementary Fig. 1). The imperfect agreement is within the variability previously observed in prints of numerous different materials[1,3] which may arise from many different factors like non-circular cross-sections, the contractile nature of the cells, or some diffusion of collagen into the jammed microgel medium during polymerization.

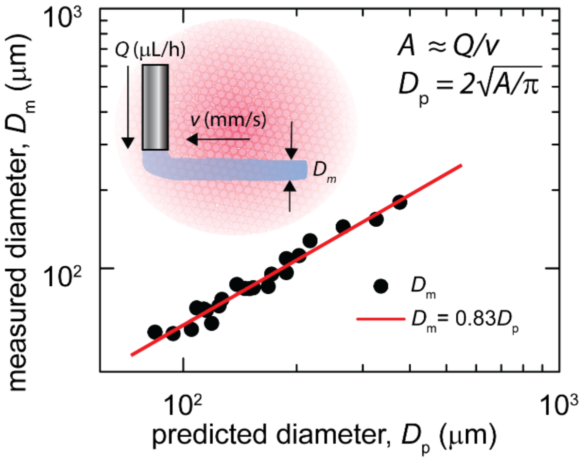

Supplementary Figure 1. We predict the feature size of a printed beam of ECM and cells based on the continuity equation, $Q = vA$, where *Q* is the volumetric flow rate of material deposition, *v* is the needle tip translation speed, and *A* is the cross-sectional area of the printed feature. Comparing the predicted diameter, D_p, to the measured average diameter, D_m, for each beam, we find that $D_m = 0.83D_p$ ($R^2 = 0.96$).

To test whether collagen gels form within the 3D printed microbeams, we perform confocal reflectance microscopy measurements to image the fibre networks. We observe networks that look qualitatively similar to those normally cast on glass slides or at the bottom of petri dishes. We

show the differences between 3D printed and cast collagen networks below in Supplementary Figure 5. Additionally, by simultaneously performing fluorescence imaging of dyed cells embedded in the collagen, we see that cells behave as they typically do in cast collagen networks, extending filapodia and interacting with the 3D printed networks (Supplementary Fig. 2).

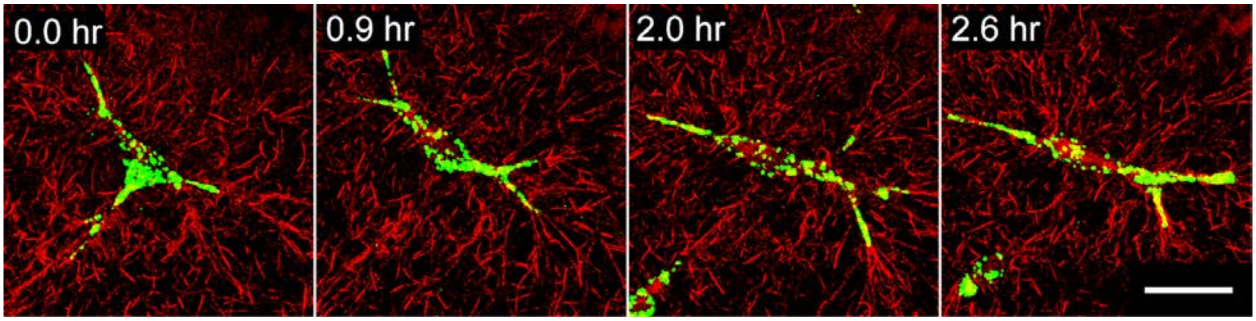

Supplementary Figure 2. After printing mixtures of collagen-1 and 3t3 fibroblasts, we see a polymerized network using confocal fluorescence microscopy with cells engaging the fibres, extending, and contracting. (Scale bar: 30 μm)

<u>Buckling Wavelength Measurements</u>

Qualitative observations of the cell-ECM microbeams reveal that beams originally printed straight develop undulations over a 24-hour period. To quantify the wavelength of the undulations and test EB beam theory, we take 3D confocal fluorescence microscopy images of the cell-ECM microbeams, using fluorescently labelled cells (see Methods). We employ three different methods of analysis to measure the wavelengths from the 3D confocal stacks: intensity autocorrelation analysis, 3D curve fitting of beam backbones, and direct visual inspection. We find consistent results with all three methods.

To determine λ when the undulation amplitude is small, we rely on an intensity autocorrelation analysis that is sensitive to regular oscillations. First, each confocal stack is filtered to remove noise using a 3D Gaussian kernel, one pixel in width. The filtered stack is rotated in 3D using image moments analysis to ensure that the long axis of the beam is parallel to the closest Cartesian axis, which we define as *z*. The filtered and rotated stack is then digitally stretched or compressed to ensure cube-shaped voxels, creating an intensity distribution, $I(x,y,z)$, where *x* and *y* correspond to the axes transverse to the beam direction. A maximum intensity projection is then taken along the y-axis which corresponds roughly to the direction of confocal scanning. From this projected image, we compute an intensity-intensity autocorrelation function using the Fourier correlation theorem, $C(X,Z) = F^{-1}[F[I(x,z)] \times F^{*}[I(x,z)]]$, where F represents the forward

Fourier transform, F^* is its complex conjugate, and F^{-1} is the inverse Fourier transform. Here we use the Fast-Fourier-Transform (FFT) function in MATLAB. While this analysis does not require normalization of the correlation functions, we divide by $C(0, 0)$ so that the function has a maximum value of 1 at its centre. We directly see the effects of beam undulation in this 2D correlation function, which we show in Supplementary Figure 3b. To measure a wavelength, we plot $C(0, Z)$ and identify the location of the first peak at finite Z, which equals the undulation wavelength, λ (Supplementary Fig. 3c).

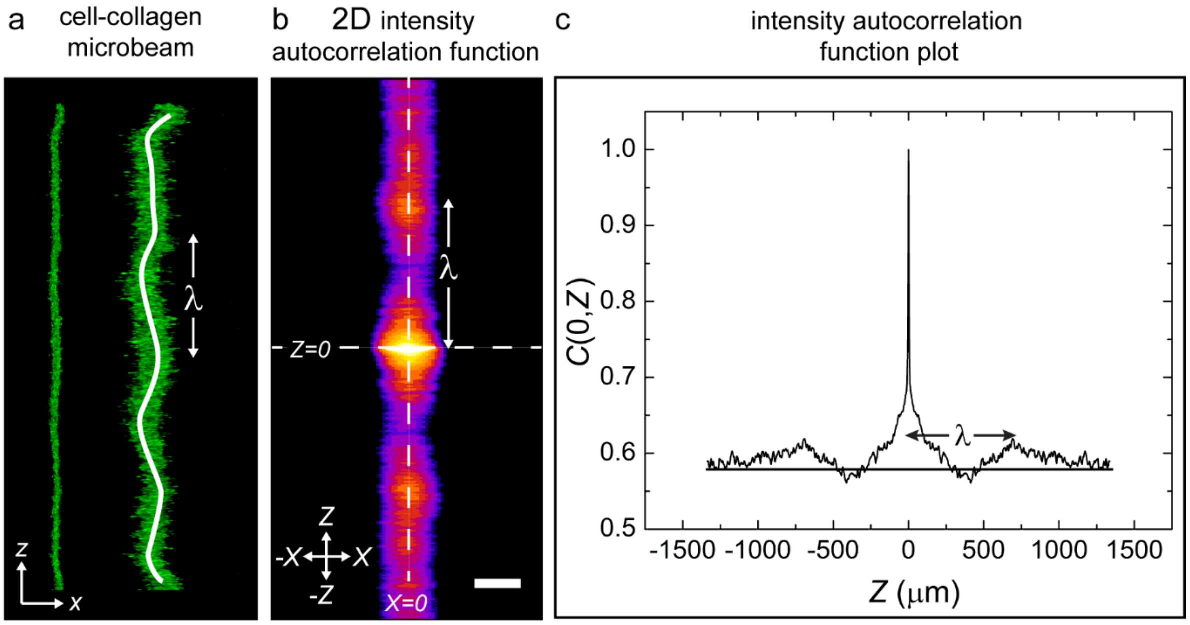

Supplementary Figure 3. (a) A 2D projection of a confocal fluorescence 3D stack shows the shape of a buckled microbeam with a small undulation amplitude. We show a digitally stretched image to accentuate the undulations (spline drawn manually to guide the eye). (b) To measure the buckling wavelength, λ, we compute a 2D intensity-intensity autocorrelation function, $C(X, Z)$. The 2D correlation function shows peaks located at $Z = \pm\lambda$. (Scale bar: 100 μm) (c) A plot of $C(0, Z)$ is used to measure λ, where the location of the first peak at finite Z is equal to λ.

To measure the undulation wavelengths using a different method for comparison, we pre-process the confocal stacks in the same way as described above, but instead of computing a correlation function, the backbone locations of the beam are measured in the *x*-*y* plane while stepping through the beam's long axis, *z*. The *x*-*y* centroids at each *z* location are found by computing the 2D centre of intensity[4]. Prior to computing the centroids, we fill in the void-space between the cells by carefully dilating and eroding the images producing a closed continuous cross-section. This step of processing is performed by direct visual inspection for each beam to

carefully choose appropriate thresholding, dilation, and erosion parameters for each beam. The resulting backbone locations are fit simultaneously to two sinusoidal functions, given by

$$x(z) = A_1 \cos(2\pi z / \lambda_1 + \phi_1) + A_2 \cos(2\pi z / \lambda_2 + \phi_2) \qquad (1)$$

and

$$y(z) = A_3 \cos(2\pi z / \lambda_1 + \phi_3) + A_4 \cos(2\pi z / \lambda_2 + \phi_4). \qquad (2)$$

We initiate the fitting procedure by visually matching the observable undulation wavelength and amplitude, λ_1 and A_1 to the backbone data; the second set of parameters, λ_2 and A_2 account for any weak bends corresponding to a λ_2 much larger than the length of the beam. While this procedure requires a lot of manual intervention, it produces measurements of λ_1 that agree with the correlation analysis described above as well as the undulations that can be observed by eye (Supplementary Fig. 4). Whether using correlation analysis or curve-fitting, all wavelengths are compared to those measured by direct visual inspection by identifying the locations of peaks and troughs in transverse-projections of the confocal fluorescence stacks; all results agree to within the spread of the data shown in Figure 3d in the manuscript body.

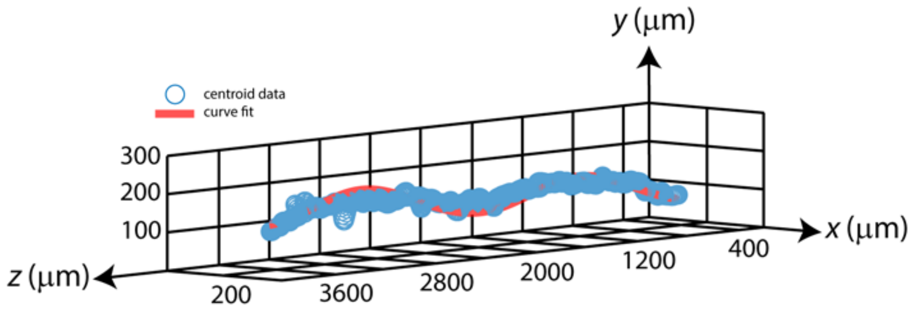

Supplementary Figure 4. The backbone locations of a buckled microbeam, measured by image centroiding (symbols), are fit by a sinusoidal function to determine the buckling wavelength. This method complements the correlation function analysis described above and produces results consistent with direct visual inspection.

<u>Collagen Microbeam Elastic Moduli</u>

To demonstrate control over collagen gelation, we perform low-amplitude oscillatory rheology on collagen gels cast between roughened 25 mm plates using an Anton-Paar MCR-702 rheometer. To mimic the solution conditions of our printing experiments, we prepare collagen-1 solutions in cell growth media supplemented with HEPES buffer (25 mM), and bring the pH to 7.4. The collagen solution is loaded into the rheometer geometry at 20 ^{o}C, and a small enclosure with a solvent trap

is put in place to prevent evaporation during testing. A CO_2 line is inserted into the enclosure to maintain 5% CO_2, and the temperature is raised from 20 ^oC to 37 ^oC at a rate of 1 ^oC/min. After 30 minutes, we apply a 1% oscillatory strain at 1 Hz and measure the elastic response, *G'*. We find that the scaling between *G'* and collagen concentration, *c*, is consistent with a $c^{2.2}$ power-law for concentration between of 0.5 mg/mL and 2.5 mg/mL, which agrees with prior work done using similar curing protocols[5]. At the lowest collagen concentrations tested, this scaling relationship breaks down, yet we find that *G'* > *G''*, indicating the formation of system-spanning networks even at low concentrations (Fig. 3c).

To test whether the collagen network shear modulus, *G'*, is the modulus relevant to the types of deformations observed in the 3D printed microbeams, we employ an alternative method to determine the elastic modulus; we 3D print cell-free collagen beams into the microgel media and manually apply axial loads while observing the response with photographic or microscopic imaging (Fig. 3a, b). The macro-beams are deformed by pressing on one end with a micro-indentation system (Hysitron Bio-soft). We find that the macro-beams buckle and then straighten-out again after the load is removed. Using the image analysis methods described above, we measure the bucking wavelength and beam-diameter; with these measurements combined with the known *G'* of the microgel medium, we are able to infer the beam elastic modulus, *E*. We extend this method to the micro-scale, performing a similar procedure on the microscope while using a printing needle to deform the microbeams (Fig. 3a, Supplementary Movie 2). Together, these tests on cell-free beams show that at the higher end of collagen concentrations, greater than 1.0 mg/mL, the elastic modulus relevant to buckling is very close to the collagen shear modulus. By contrast, at collagen concentrations of 1.0 mg/mL and below, we see that the datasets diverge (Fig. 3c). Since we are unable to measure *E* at concentrations below 0.5 mg/mL using this method, any experiments using collagen below this concentration are excluded from analysis if knowledge of *E* is required.

To investigate the differences between *G'* and *E* at low collagen concentrations, we image collagen networks at various concentrations, both cast on glass slides without surrounding microgel media and printed into the microgel medium. From laser-scanning confocal reflectance microscopy images, we find that collagen networks embedded in microgel medium look qualitatively similar to those cast as free liquid drops but have a visibly larger mesh-size at the

lower concentrations of 0.25 mg/mL and 0.5 mg/mL. Since free collagen molecules can diffuse into the surrounding microgel medium during polymerization, it is possible that the competing rates of polymerization and diffusion favour diffusion at very low collagen concentrations, resulting in a sparser network than expected. While detailed investigations are needed to determine the underlying mechanism responsible for the differences between *G'* and *E*, our mechanical measurements and reflectance images produce consistent results. Moreover, all the analyses throughout this manuscript employ the measurements of *E*, avoiding the need for a theoretical model or detailed understanding of the polymerization process. The consistency in rheology and apparent network structure also indicates that the potential roles of viscoelasticity and network complexity may contribute comparably to both sets of measurements. We would expect viscous stresses to dominate at high shear rates; given that cell-driven beam buckling is a very slow process, we believe viscous stresses are likely small compared to elastic stresses or shear-rate independent yielding of microgels. Over long time scales the non-elastic contributions would likely arise from network remodeling and reorganization, which we have explored in multiple different control experiments described throughout the manuscript.

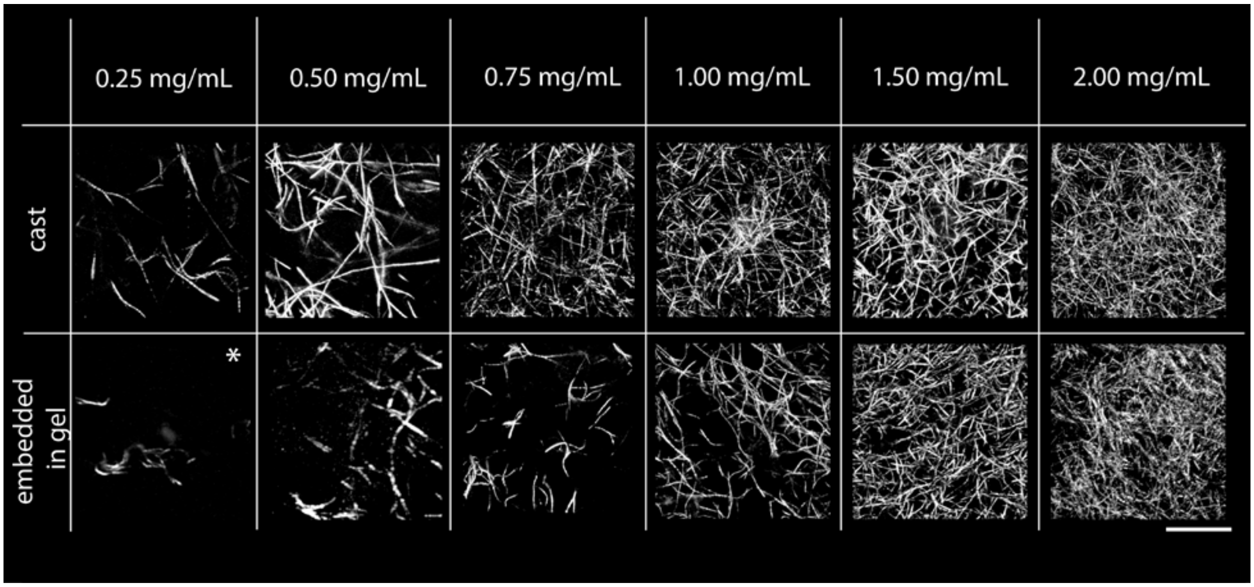

Supplementary Figure 5. Confocal reflectance microscopy is used to qualitatively compare the network structures exhibited by cast and 3D printed collagen networks. At high concentrations, the two types of sample appear to have similar structures, while they deviate visibly at low concentrations. These observations are consistent with the deviations seen in rheological measurements (Fig. 3c). (* denotes that this sample is displayed at 1/3 the magnification of all other samples in the figure due to the very sparse fibre density). (Scale bar: 25 μm)

We use only measured quantities to test the applicability of EB theory to cell-driven buckling; λ is predicted using measurements of *E* from cell-free collagen beams, combined with

measurements of G', and R. Finding excellent agreement, we extend this approach to cell-ECM microbeams, using EB theory to determine E from measurements of λ. Using 57 measurements of λ from cell-ECM microbeams containing 3t3, GL261, and Panc02 cells, we compute the beam elastic modulus containing cells, E^+, given by

$$E^+ = \left(\frac{\lambda}{2\pi}\right)^4 \frac{G'}{I}, \qquad (3)$$

and plot the results versus the cooresponding beam elastic modulus without cells, E^-, measured as described above (Supplementary Fig. 6). The 4th power dependence of E on λ will lead to large variations in E^+ and E^- arising from small variations in λ, as observed in this plot. However, these datapoints span several decades in both directions, revealing a linear relationship given by $E^+ = 1.13\ E^-$. We use this relationship to determine E^+ in calculations of σ_{cell} where no λ is measured (break-up threshold and axial contraction datapoints), converting from E^- to E^+. In experiments where beams buckle and λ is measured, we directly solve for E^+ without using E^- when calculating cell generated stress, σ_{cell} (see manuscript body describing models involving σ_{cell}).

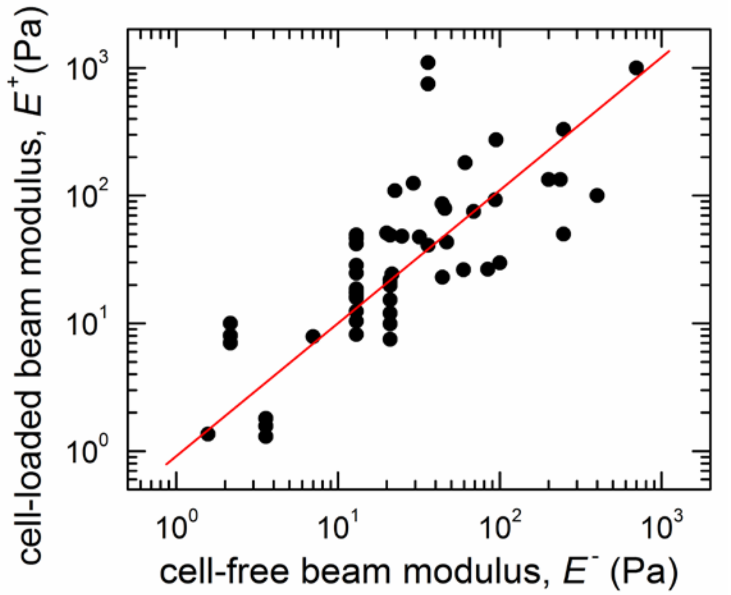

Supplementary Figure 6. We 3D print microbeams having different collagen concentrations and measure their elastic moduli with and without embedded cells (E^+ and E^-). In both cases, the buckling wavelength, λ, is used to determine the beam elastic modulus at a given collagen concentration. While the 4th power dependence of E on λ generates large variations in E from small variations in λ, the large range of moduli measured shows a linear scaling, given by $E^+ = 1.13\ E^-$.

Recent investigations of collagen gel elasticity have shown these networks having strong non-linear responses to compression and stretch[6,7]. For example, the shear modulus increases strongly in stretched networks and decreases weakly in compressed networks. Correspondingly, the modulus of uniaxial strain increases strongly with stretch along the same axis and decreases weakly with compression. One major outcome of these observations is the discovery of a highly asymmetric Poisson's ratio about the unstrained state. While these strong nonlinearities have major implications in the mechanical performance of collagen networks in vitro and in vivo, it is unclear how they may contribute to the mechanics of the cell-ECM microbeams investigated here. For example, when cells within the microbeams contract they probably initially tense the collagen networks, suggesting that stiffening may occur. However, in all cases in which microbeams buckle, fail, or contract, the net effect is to contract the network; the pliable microgel environment allows the entire collagen network to compress, suggesting a weak strain softening could be occurring. We hope that our results inspire new investigations of collagen elasticity in contexts where the network boundary conditions evolve in time. We believe that using jammed microgels as encasing environments for biopolymer networks represents a path forward for such studies with tuneable boundary conditions.

Wavelength measurements broken down by cell type and test type

We compare various measurements of λ to predictions from EB theory, using the classic result, $\lambda = 2\pi(EI/G')^{1/4}$. Here we determine E, I, and G', from experimental measurements described above and in the manuscript body. For the three cell types investigated here, and for cell-free beams under manual loading, we find that EB theory predicts our data with no fitting parameters (R^2 = 0.93, Supplementary Fig. 7). Control experiments without cells exhibit no spontaneous buckling, indicating that undulations emerging in cell-ECM microbeams are a form of buckling driven by the contractile stresses generated by the cells within.

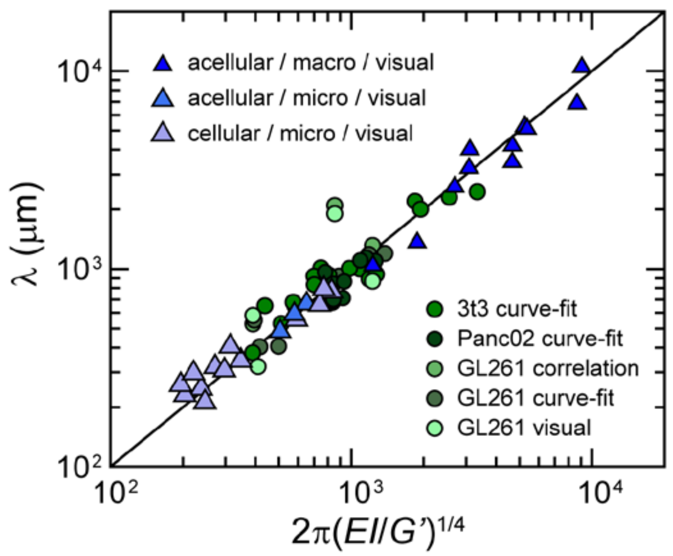

Supplementary Figure 7. Euler-Bernoulli (EB) beam theory predicts the relationship between wavelength, λ, and beam properties *E*, *I*, and *G'*, for multiple cell types, cell-free beams, and multiple wavelength measurement methods. Errorbars represent standard deviations about mean values from repeated measurements. Wavelengths measured by visual inspection for manually bucked beams (*n* = 11 acellular macrobeams, *n* = 3 acellular microbeams, *n* = cellular microbeams). For cell-driven buckling, multiple types of wavelength measurements were performed (*n* = 8 Panc02 cells analysed by curve-fit, *n* = 20 3t3 cells analysed by curve-fit, *n* = 6 GL261 cells analysed by curve-fit, *n* = 4 GL261 cells analysed by correlation analysis, and *n* = 4 GL261 cells analysed by visual inspection).

Elastic Shear Modulus and Yield Stress of Microgel Medium

To characterize the material properties and yielding behaviour of the mircrogel media used here, we perform both oscillatory and unidirectional shear rheology on samples prepared at several microgel concentrations (2.2% to 10%) using a Malvern Kinexus rheometer equipped with a roughened cone on plate geometry. Within the oscillatory frequency range of 10^{-3} – 10^{2} Hz, we find that *G'* is greater than *G''* for all polymer concentrations, indicating that the samples are dominantly solid-like (Supplementary Fig. 8a, b). Choosing 1 Hz as a representative frequency, we plot *G'* versus microgel concentration, *c*, finding a scaling relationship consistent with $G' \sim c^{9/4}$, as previously reported for the same microgels prepared in water[8]. To measure the yield stress of these microgel samples, we perform unidirectional shear tests described previously[1,2,8,9]. We find the experimental relationship between stress and shear-rate is well described by the Hershel-Bulkley model, given by $\sigma = \sigma_{\mathrm{y}}(1+(\dot{\gamma}/\dot{\gamma}_{\mathrm{c}})^{\mathrm{p}})$, where σ is applied stress, σ_{y} is yield stress, $\dot{\gamma}$ is shear rate, $\dot{\gamma}_{\mathrm{c}}$ is the crossover shear-rate between solid-like and fluid-like behaviours, and p is a dimensionless number of order 0.5 (Supplementary Fig. 8c)[10]. We determine σ_{y} for each sample by fitting the Hershel-Bulkley model to the data, finding the scaling relationship between σ_{y} and

c consistent with $\sigma_y \sim c^{9/4}$, just as found with the G' data (Supplementary Fig. 8d). Plotting σ_y versus G' reveals an excellent correlation between the two variables, in very good agreement with our previous findings that $\sigma_y = 0.13\ G'$, which we found to arise from the elastic deformation energy required to drive single-particle rearrangements during shearing (Supplementary Fig. 8e).

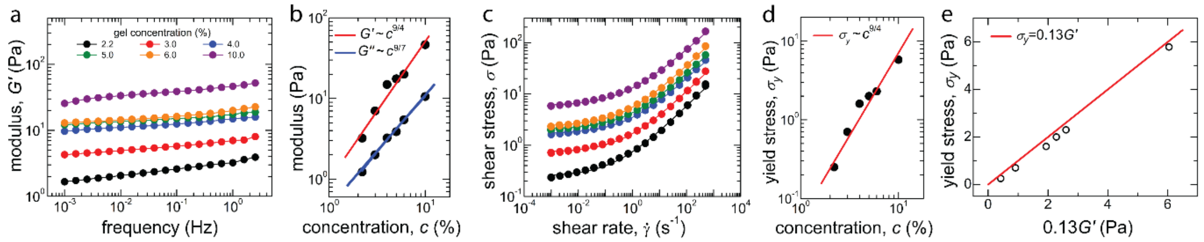

Supplementary Figure 8. (a) The storage shear modulus, *G'*, of microgel media rises with microgel concentration and exhibits a weak frequency dependence. (b) The increase in *G'* with microgel concentration is accompanied by an increase in the loss modulus, *G"*; the loss modulus remains below the storage modulus for all concentrations indicating solid-like behaviour. (c) Unidirectional shear rheology indicates the transition from solid-like behaviour to fluid-like behaviour with increasing shear rate; the measured shear stress approaches a plateau as the shear rate decreases, which corresponds to the yield stress, σ_y. (d) Examining the relationship between concentration and yield stress, we see that $\sigma_y \sim c^{9/4}$. (e) The relationship between *G'* from (b) and the yield stress from (d) is well described by $\sigma_y = 0.13G'$.

Microscopy at the microgel-collagen interface

To examine the interface between collagen microbeams and the surrounding microgel medium, we print 1.9 mg/mL collagen-1 solutions into 0.15% (w/w) microgel medium. To image the microgel medium, we disperse 4 μm diameter fluorospheres which act as fiducial markers. Imaging with laser scanning confocal fluorescence microscopy, we examine y-z cross-sections by integrating the intensity from a 3D stack along the beam's long axis (the x-axis). From these y-z projections, we see a circular void that shows where the microgels have been displaced by the deposited collagen. To quantify the sharpness of the interface between collagen and microgels, we examine the x-y slice through the beam's mid-plane. By integrating the intensity of this x-y slice along the x-axis, we see how particle density rises from within the interface and plateaus in the bulk, far from the beam. This intensity profile rises from 0 to its plateau value over a distance of about 25 μm. Since these microgels are approximately 4-5 μm in diameter, we expect intermixing between collagen and several layers of microgels to occur within this zone.

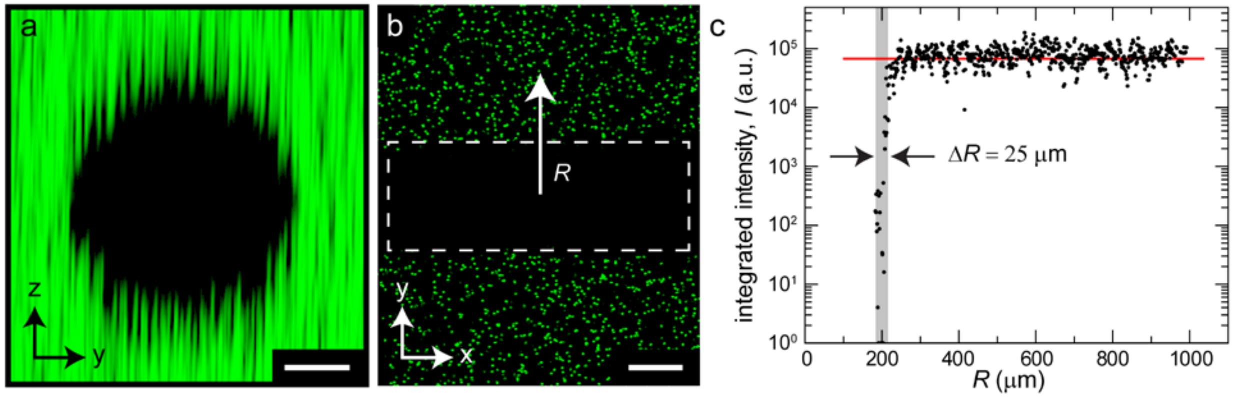

Supplementary Figure 9. (a) End-on slices through confocal microscopy stacks of printed collagen beams within the microgel medium(green tracer particles) reveal a strikingly circular cross-section and no mixing between the microgel phase and the collagen network outside of a surface-zone. (Scale bar: 100 μm) (b) Top-view slices through the mid-plane of these beams shows the same exclusion of the microgel phase from the printed collagen zone (dashed line). (Scale bar: 200 μm) (c) The integrated intensity profile of the image in (b), measured from the central axis of the beam, shows a sharp rise from 0 to a plateau in the microgel bulk. The width of the intermixing zone between collagen and microgels is found to be approximately 25 μm.

Length-dependence of microbeam contraction

To test whether friction may dominate beam contraction in a long-beam limit, we print cell-ECM beams of different lengths (1.0 mg/mL collagen) into microgel media with a yield stress of 1.9 Pa. This combination of parameters lays in the contraction regime displayed in Figure 4b. Like the 10 mm long beams described in the manuscript body, we find that short beams, 1 mm in length, also contract. By contrast, much longer beams, 30 mm in length, do not contract at all. We find that the beam appears to compact radially and becomes smoother at its surface over a 24 hour delay between measurements, yet identifiable features do not appear to move at all along the axial direction. Thus, a friction-dominated limit appears to emerge with increasing beam length, which we hope to explore further in future work (Supplementary Fig. 10).

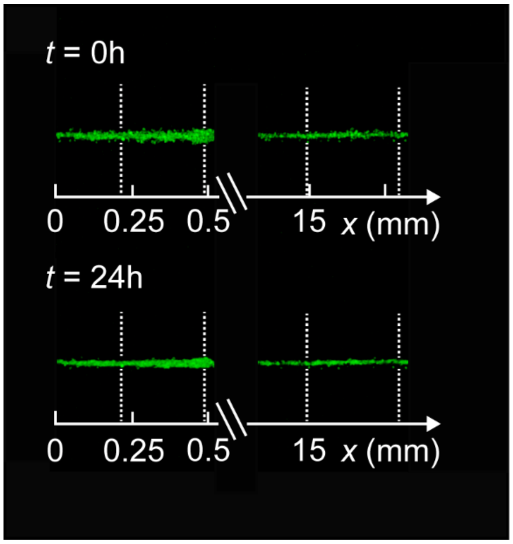

Supplementary Figure 10. Sections of a 30 mm long beam are shown just after printing (t = 0 h) and one day after printing (t = 24 h). Images were collected with confocal fluorescence microscopy; the cells in the microbeam are dyed green. While the beam appears to compact radially and attain a smoother surface with time, features do not appear to move at all along the axial direction. Irregularities in beam shape and individual cells can be identified at the same locations along the beam at the two different time points (vertical dashed lines provided to link chosen features to axis labels). To show that this stability is persistent along the beam length, we show two sections separated by approximately 15 mm.

Cell Volume Fraction Measurements

We approximate the cell volume fraction, ϕ, within each microbeam as $\phi \approx V / R_{\mathrm{nn}}^{3}$, where V is the average cell volume and R_{nn} is the nearest-neighbour center-to-center cell spacing. This estimate ignores geometrical factors associated with the details of cell distribution. We measure the average cell volume from thresholded confocal fluorescence stacks measured at high magnification with a 60X oil immersion objective, finding $V \approx 2073 \pm 603\ \mu\mathrm{m}^3$ (mean ± standard deviation, N = 19 cells) for 3t3 cells and $V \approx 3325 \pm 1000\ \mu\mathrm{m}^3$ (N = 20 cells) for GL261 cells. To measure R_{nn}, we employ an autocorrelation method similar to that described above for measuring the undulation wavelength. Instead of analysing $C(X,Z)$ from a projection of data-stacks into 2D, we compute a 3D spatial correlation function, $C(X,Y,Z)$, from the processed 3D stacks, $I(x, y, z)$. We then compute an orientational average of $C(X,Y,Z)$ over the 4π steradian solid angle in polar coordinates, producing a correlation function, $C(R)$, that varies with $R = \sqrt{X^2 + Y^2 + Z^2}$. Over short distances, this correlation function is sensitive to the intensity variations associated single cell shapes, decaying approximately like $R^{-1.95}$ following a plateau approximately one cell diameter in width (Supplementary Fig. 9a). Oscillations in $C(R)$ are seen about the decaying power-law;

plots of $C(R) \times R^{1.95}$ exhibit oscillations at longer length-scales that arise from cell-cell spatial correlations (Supplementary Fig. 9b). By measuring the location of the first peak in these plots, we determine the average nearest-neighbour distance, R_{nn}, between cells in the microbeams. For the lower volume fraction beams, with $\phi < 5\%$, we can validate this procedure by visually counting the number of cells and measuring the dimensions of beams with ImageJ, finding agreement in volume fraction to within a factor of two. Additionally, R_{nn} measurements were checked visually using ImageJ by identifying nearest neighbours and measuring their centre-to-centre distances by eye, finding agreement to within 31%. These errors in measurements of linear dimension and volume fraction are consistent with one another; an error of nearly 30% in a linear dimension will lead to roughly a factor of two error in volume approximations. While a higher degree of certainty is desirable, since the data span multiple decades in parameter space, factors of two in either direction do not change the principle conclusions.

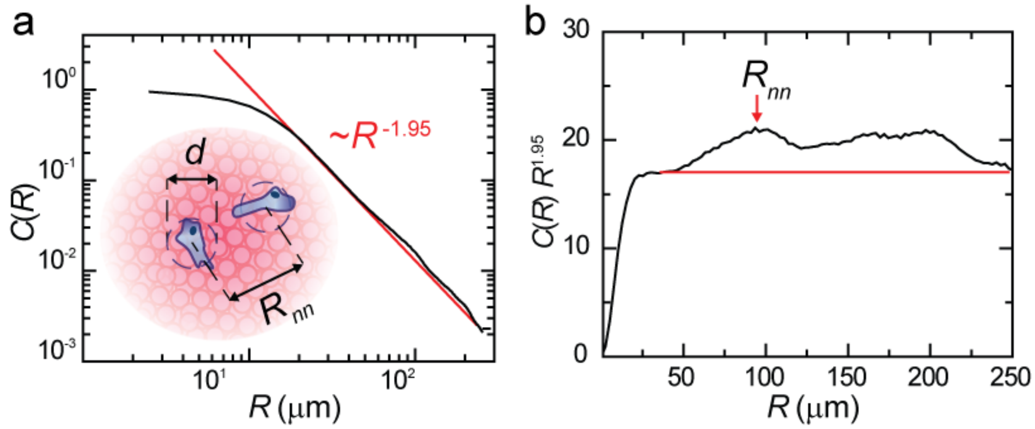

Supplementary Figure 11. (a) The nearest neighbour distance between cell pairs, R_{nn}, is measured by computing the intensity-intensity autocorrelation function, $C(R)$, of confocal stack intensity distributions. We find that $C(R)$ resembles a modified Lorentzian distribution decaying to a power of 1.95. (b) Multiplying $C(R)$ by $R^{1.95}$ removes the decay and accentuates long-wavelength oscillations that arise from cell-cell spatial correlations. We use the location of the first peak in this plot to determine R_{nn}.

Literature data points

The datapoints shown in Figure 5b labelled "2D literature" were taken from published manuscripts describing traction force microscopy measurements of cells on soft, flat, 2D substrates[11-13]. In reference 11, bovine aortic endothelial cells were used on polyacrylamide substrates with Young's moduli of 1, 2.5, 5 and 10 kPa; mean traction stresses of 200, 250, 300 and 375 Pa were reported, respectively. In reference 12, NIH 3t3 fibroblasts were used on polyacrylamide substrates with shear moduli of 3, 6, 8, 16 and 30 kPa; mean traction stresses of 250, 300, 320 and 550 and 525

Pa were reported, respectively. In reference 13, NIH 3t3 fibroblasts were used on polyacrylamide substrates with Young's moduli of 14 and 30 kPa; mean traction stresses of 620 and 1090 Pa were reported, respectively. We treat these measurements as the 2D equivalent of the cell generated stress estimated from our data and plot them without modification onto our graph of cell-generated stress in 3D (Fig. 6b). We find that a scaling law fit to our data in 3D extrapolates through this 2D traction stress data to within a factor of approximately 2. Additionally, recent investigations of cell-generated traction forces in 3D matrices agree very well with our measurements, laying close to the extrapolated fit to our data-points [14-16]. From the traction forces distributions and strain energies measured using 3D TFM methods in the literature, we estimate the cell-generated stress (Supplementary Fig. 12). For example, reference 14 reports a strain energy of 1 pJ in a 2.4mg/mL collagen-1 network of modulus 118 Pa. From this strain energy we predict an average traction stress of 210 Pa. Ref 15 reports 0.1 μN traction forces in a Matrigel network with a modulus of 90 Pa. In this case we estimate an average traction stress of 162 Pa. Similarly, ref. 16 reports a strain energy of 0.1 pJ in a 1.2mg/mL collagen-1 network of modulus 1645 Pa. We estimate an average traction stress of 250 Pa. Cell volumes are made using to the data reported in the manuscripts and are found to be between 1000 – 5000 μm^3, within the range of the 2000 - 3000 μm^3 we measure in our experiments.

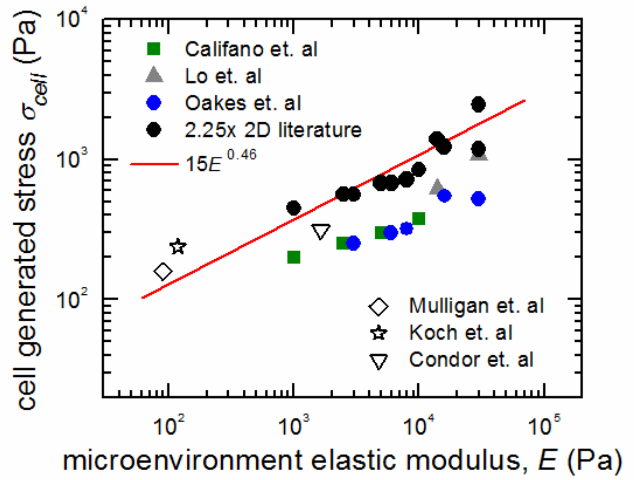

Supplementary Figure 12. We surveyed the literature for measurements of traction-stresses exerted by cells on 2D elastic substrates. A scaling law fit to our 3D data extrapolates to the 2D data when rescaled by a factor of 2.25. Three-dimensional traction force microscopy literature were also surveyed. Shown in open symbols, we establish that these data also fall on our prediction of cell generated stress relative to microenvironmental modulus.

3D Printed Neural Crest Model

To test our 3D bioprinting method's ability to generate a model developing tissue made from multiple cell types or materials, we designed a 3D analogue of textbook-level illustrations of the developing neural crest. Fetal neural development is typically depicted as a series of snapshots showing the formation of the neural tube through the buckling of the neural plate and the translocation of the neural plate border and epidermis. In such a time-series, the epidermal sheet converges to cover the neural tube. To demonstrate our method's capabilities we 3D print structures made from fluorospheres (Fig. 7). The same structure can be printed from living cells, though we recognize that mimicking morphogenesis in vitro using such structures is beyond our current capabilities. To fabricate the 3D models, we fill three separate wells with solutions of fluorospheres of different colours. We program our printing stage to visit each separate well and draw an arbitrary volume of fluorosphere solution into a syringe. The different fluorosphere solutions are printed one at a time; when changing from one colour to the next, the syringe is programmed to empty into a waste well and wash itself with clean water and then clean microgels. This method allows the printing of one seamless structure out of three different materials with a single set of printing commands. The green beads represent the epidermis, the blue beads represent the neural plate border, and the red beads represent the neural plate. We envision that the ability to fabricate these structures from living cells will one day enable testing a watchmaker's approach to tissue engineering, in which structures at each stage of development are created and allowed to further mature, with the goal of discovering how to set in motion later stages of development from conditions mimicking earlier stages.

Works cited

1 Bhattacharjee, T. *et al.* Writing in the granular gel medium. *Science advances* **1**, e1500655 (2015).

2 Bhattacharjee, T. *et al.* Liquid-like solids support cells in 3D. *ACS Biomaterials Science & Engineering* **2**, 1787-1795 (2016).

3 O'Bryan, C. S. *et al.* Three-dimensional printing with sacrificial materials for soft matter manufacturing. *MRS Bull.* **42,** 571–577 (2017).

4 Burger, W. & Burge, M. J. *Principles of Digital Image Processing*, Springer-Verlag London Limited (2009).

5 Yang, Y. L. & Kaufman, L. J. Rheology and confocal reflectance microscopy as probes of mechanical properties and structure during collagen and collagen/hyaluronan self-assembly. *Biophys. J.* **96,** 1566–1585 (2009).

6 Van Oosten, A. S. *et al.* Uncoupling shear and uniaxial elastic moduli of semiflexible biopolymer networks: compression-softening and stretch-stiffening. *Scientific reports* **6**, 19270 (2016).

7 Steinwachs, J. *et al.* Three-dimensional force microscopy of cells in biopolymer networks. *Nature methods* **13**, 171 (2015).

8 Bhattacharjee, T. *et al.* Polyelectrolyte scaling laws for microgel yielding near jamming. *Soft Matter* **14,** 1559–1570 (2018).

9 O'Bryan, C. S. *et al.* Self-assembled micro-organogels for 3D printing silicone structures. *Sci. Adv.* **3,** e1602800 (2017).

10 Herschel, W. H. & Bulkley, R. Konsistenzmessungen von Gummi-Benzollösungen. *Kolloid Z.* **39**, 291–300 (1926).

11 Califano, J. P. & Reinhart-King, C. A. Substrate stiffness and cell area predict cellular traction stresses in single cells and cells in contact. *Cell. Mol. Bioeng.* **3**, 68–75 (2010).

12 Oakes, P. W., Banerjee, S., Marchetti, M. C. & Gardel, M. L. Geometry regulates traction stresses in adherent cells. *Biophysical journal* **107**, 825-833 (2014).

13 Lo, C.-M., Wang, H.-B., Dembo, M. & Wang, Y.-l. Cell movement is guided by the rigidity of the substrate. *Biophysical journal* **79**, 144-152 (2000).

14 Koch, T. M., Münster, S., Bonakdar, N., Butler, J. P. & Fabry, B. 3D traction forces in cancer cell invasion. *PloS ONE* **7**, e33476 (2012).

15 Mulligan, J. A., Feng, X. & Adie, S. G. Quantitative reconstruction of time-varying 3D cell forces with traction force optical coherence microscopy. *Scientific reports* **9**, 4086 (2019).

16 Cóndor, M. *et al.* Breast cancer cells adapt contractile forces to overcome steric hindrance. *Biophysical journal* **116**, 1305-1312 (2019).
