# Supplementary material for: Quantitative characterization of 3D bioprinted structural elements under cell generated forces
Source: Nat Commun. 2019 Jul 10;10:3029. doi: 10.1038/s41467-019-10919-1 (PMC6620298; doi:10.1038/s41467-019-10919-1)
Supplement: Supplementary file 3 — Reporting Summary [file 41467_2019_10919_MOESM3_ESM.pdf]

nature research

Corresponding author(s): DBPR: NCOMMS-19-12315-T

Last updated by author(s): May 15, 2019

Reporting Summary

Nature Research wishes to improve the reproducibility of the work that we publish. This form provides structure for consistency and transparency in reporting. For further information on Nature Research policies, see Authors & Referees and the Editorial Policy Checklist.

Statistics

For all statistical analyses, confirm that the following items are present in the figure legend, table legend, main text, or Methods section.

n/a	Confirmed	
☐	☒	The exact sample size (*n*) for each experimental group/condition, given as a discrete number and unit of measurement
☐	☒	A statement on whether measurements were taken from distinct samples or whether the same sample was measured repeatedly
☒	☐	The statistical test(s) used AND whether they are one- or two-sided<br>*Only common tests should be described solely by name; describe more complex techniques in the Methods section.*
☐	☒	A description of all covariates tested
☐	☒	A description of any assumptions or corrections, such as tests of normality and adjustment for multiple comparisons
☐	☒	A full description of the statistical parameters including central tendency (e.g. means) or other basic estimates (e.g. regression coefficient) AND variation (e.g. standard deviation) or associated estimates of uncertainty (e.g. confidence intervals)
☒	☐	For null hypothesis testing, the test statistic (e.g. *F*, *t*, *r*) with confidence intervals, effect sizes, degrees of freedom and *P* value noted<br>*Give P values as exact values whenever suitable.*
☒	☐	For Bayesian analysis, information on the choice of priors and Markov chain Monte Carlo settings
☒	☐	For hierarchical and complex designs, identification of the appropriate level for tests and full reporting of outcomes
☒	☐	Estimates of effect sizes (e.g. Cohen's *d*, Pearson's *r*), indicating how they were calculated

Our web collection on statistics for biologists contains articles on many of the points above.

Software and code

Policy information about availability of computer code

Data collection	NIS Elements 4.20
Data analysis	MatLab R2018a<br>OriginPro 8.5<br>FIJI

For manuscripts utilizing custom algorithms or software that are central to the research but not yet described in published literature, software must be made available to editors/reviewers. We strongly encourage code deposition in a community repository (e.g. GitHub). See the Nature Research guidelines for submitting code & software for further information.

Data

Policy information about availability of data

All manuscripts must include a data availability statement. This statement should provide the following information, where applicable:

- Accession codes, unique identifiers, or web links for publicly available datasets
- A list of figures that have associated raw data
- A description of any restrictions on data availability

Data supporting the findings of this manuscript are available from the corresponding author upon reasonable request. A reporting summary for this Article is available as a Supplementary Information file.

The source data underlying Figs. 3c-d, 5b and Supplementary Figs. 7 are provided as a Source Data file.
Data supporting the findings of this manuscript are available from the corresponding author upon reasonable request. A reporting summary for this Article is available as a Supplementary Information file.

The source data underlying Figs. 3c-d, 5b and Supplementary Figs. 7 are provided as a Source Data file.

Field-specific reporting

Please select the one below that is the best fit for your research. If you are not sure, read the appropriate sections before making your selection.

☒ Life sciences ☐ Behavioural & social sciences ☐ Ecological, evolutionary & environmental sciences

For a reference copy of the document with all sections, see nature.com/documents/nr-reporting-summary-flat.pdf

Life sciences study design

All studies must disclose on these points even when the disclosure is negative.

Sample size	Each of the 69 data-point displayed in Figure 3D corresponds to a single measurement (n = 1). This sample size was chosen because the statistically meaningful observation is the trend that spans many decades of parameter space, while binning these data and reporting averages only obscures the trend formed by the numerous data points. The data points displayed in Figure 4b represent n = 3 samples which all exhibited the behavior denoted by the figure legend. The data points displayed in Figure 5a and 5b correspond to single measurements (n = 1). These data-points were not binned and averaged because the statistically meaningful observations were the changes observed over several decades of parameter space; grouping these data-points into averaged bins would obscure the corresponding trends and boundaries. The practices followed here are standard in the types of in vitro experimental biophysics study performed here.
Data exclusions	When 3D printing structures made from cells and ECM, the quality of material deposition is occasionally inadequate for experimentation; highly inhomogeneous beams with large breaks or clumps of material cannot be analyzed. Thus, when these imperfections were encountered, the samples were discarded. Additionally, some data-points in Figure 5a were labeled "indeterminate" because cell density was excessively low to allow analysis.
Replication	In all quantitative data displayed in the manuscript figures, reproducibility was verified by systematically varying one independent variable at a time and observing the resulting trend. As the variations between different measurements span several decades of parameter space and follow well-defined trends, we believe the data demonstrate good reproducibility. For the qualitative categorizations reported in Figure 4b, each data-point corresponds to n = 3 replicates, all of which exhibited the same behavior.
Randomization	The samples were not prepared and tested in a sequentially ordered manner; since the phenomenon observed is the first of its kind, experimental parameters were guessed and tested. These parameters include collagen concentration, microgel concentration, beam diameter, and cell density. As coarse trends emerged over the large span of variable space explored, additional samples were designed in a random order to fill in missing gaps in trends.
Blinding	Blinding is not relevant to the type of in vitro biophysics study performed here. As the phenomena discovered in this investigation are new and unanticipated, the researchers were not susceptible to biases typical of studies requiring blinding measures.

Reporting for specific materials, systems and methods

We require information from authors about some types of materials, experimental systems and methods used in many studies. Here, indicate whether each material, system or method listed is relevant to your study. If you are not sure if a list item applies to your research, read the appropriate section before selecting a response.

Materials & experimental systems

n/a	Involved in the study
☒	☐ Antibodies
☐	☒ Eukaryotic cell lines
☒	☐ Palaeontology
☒	☐ Animals and other organisms
☒	☐ Human research participants
☒	☐ Clinical data

Methods

n/a	Involved in the study
☒	☐ ChIP-seq
☒	☐ Flow cytometry
☒	☐ MRI-based neuroimaging

Eukaryotic cell lines

Policy information about cell lines

Cell line source(s)	3t3: ATCC<br>GL261: NCI<br>PAN 02: NCI
Authentication	ATCC and NCI authenticate all their cells using publicly available methods.

Mycoplasma contamination	GL261 and PAN 02 were tested by NCI for mycoplasma contamination upon their deposition into the cell bank. No cells used in this study were tested for mycoplasma contamination within our laboratories.
Commonly misidentified lines<br>(See ICLAC register)	n/a
