# Supplementary material for: Quantitative characterization of 3D bioprinted structural elements under cell generated forces
Source: Nat Commun. 2019 Jul 10;10:3029. doi: 10.1038/s41467-019-10919-1 (PMC6620298; doi:10.1038/s41467-019-10919-1)
Supplement: Supplementary file 4 — Description of Additional Supplementary Files [file 41467_2019_10919_MOESM4_ESM.pdf]

Description of Additional Supplementary Files

Supplementary Movie 1: Microscopic imaging of cells contracting collagen in jammed microgels.

Mouse 3t3 fibroblasts fluorescently dyed with cell tracker green, mixed with collagen-1 solution, then printed into the jammed microgel medium. Confocal microscopy shows the cells contract and deform the collagen-1 fiber network.

Supplementary Movie 2: Microscopic imaging of cell-collagen beam buckling in jammed microgel.

Mouse 3t3 fibroblasts fluorescently dyed with cell tracker green and mixed with collagen-1 are 3D printed into the jammed microgel medium. The beam is imaged with confocal microscopy, showing the beam buckle. The image is digitally stretch to assit visual observation of buckling features.

Supplementary Movie 3: Microscopic imaging of cell-collagen beam failure in jammed microgel.

Mouse GL261 cells expressing GFP fluoresce are mixed with and collagen-1 and 3D printed into the jammed microgel medium. Confocal microscopy shows the beam failing and breaking into small pieces over time.

Supplementary Movie 4: Microscopic imaging of cell-collagen beam contraction in jammed microgel.

Mouse 3t3 fibroblasts fluorescently dyed with cell tracker green and mixed with collagen-1 are 3D printed into the jammed microgel medium. Confocal microscopy shows the beam axially contracting over time.
